# In their own words: qualitative interviews with veterinarians on handling decisions during dog examinations

**DOI:** 10.3389/fvets.2026.1761014

**Published:** 2026-03-20

**Authors:** Lindsay Nakonechny, Katy Schroeder, Anastasia Chiara Stellato

**Affiliations:** 1 Department of Animal and Food Sciences, Texas Tech University, Lubbock, TX, United States; 2 Department of Counselor Education, University of Iowa, Iowa City, IA, United States

**Keywords:** clinical practice, handling decisions, professional wellbeing, stress-reducing practices, veterinary perceptions

## Abstract

**Introduction:**

Routine veterinary visits can be a major source of fear and stress in dogs, creating welfare concerns and safety risks for veterinary teams and owners. Although stress-reducing practices have been widely promoted, limited research has examined how veterinarians describe their handling decisions and the factors that shape them in everyday practice. This qualitative study aimed to (1) explore how veterinarians in Canada and the United States conduct routine dog physical examinations; (2) identify clinic-, patient-, client-, and veterinarian-related factors, including professional well-being, that influence handling decisions; and (3) examine veterinarians’ perceptions of stress-reducing practices, including perceived benefits and challenges.

**Methods:**

Virtual semi-structured individual interviews were conducted with 17 veterinarians who provide clinical care to dogs between November 2024 and February 2025. Participants were recruited using snowball and convenience sampling. Transcripts were analyzed using inductive content analysis within an interpretivist paradigm.

**Results:**

Four major categories and 11 sub-categories emerged from the data: (1) approaches to dog handling; (2) factors affecting handling approaches; (3) veterinarian professional well-being; and (4) perceptions of stress-reducing practices.

**Discussion:**

Handling during routine dog physical examinations reflects negotiation between patient needs, safety, workplace constraints, and veterinarian wellbeing, with stress-reducing practices valued and utilized, yet not perceived to be uniformly feasible across situations. Clinic-level support (team training, supportive management, scheduling) and attention to veterinarians’ professional well-being may promote more consistent use of stress-reducing handling, improving dog welfare, safety, and owner experiences.

## Introduction

Routine veterinary visits are often stressful for dogs and owners, with many dogs showing fear that can impede handling and compromise examinations (1–5). Fear can escalate to aggression (5, 6) which poses injury risk to the veterinary team and animal patient. Bite and scratch injuries from animal patients are among the most common injuries to veterinarians (7, 8). Thus, dog fear is not only an animal welfare concern, but it also creates risks and challenges for veterinary staff. Clients are also affected by their dog’s stress during examinations; owners report lower willingness to return after stressful visits (9), express concern about restraint and their pet being taken out of the examination room (10) and prefer less physical restraint if their dog is fearful (11). Despite the impacts of veterinarian-dog interactions on animal patients and owners, limited research has examined the handling practices of veterinarians and what factors inform such decisions.

Preventing and reducing fear in veterinary settings has become increasingly important among veterinary staff and clinics. Stress-reducing practices have emerged that are aimed at preventing and alleviating fear, anxiety, and stress in animal patients during routine care (6, 12). These practices include, but are not limited to: using only minimally necessary levels of physical restraint, performing the examination where the dog is most comfortable, allowing time to acclimate to the clinic room prior to starting the examination, adjusting handling based on the dog’s body language, providing positive experiences or distractions (e.g., treats), using pre-visit medications to reduce arousal (e.g., trazodone), and using behavior modification strategies, such as counterconditioning and/or desensitization (6, 12). In an intervention study, dogs undergoing veterinary visits with stress-reducing strategies, such as the provision of treats, use of anti-slip mats, and little to not restraint was associated with reduced dog behavioral and physiological indicators of stress across several appointments, compared to controls (13, 14). Another experimental study also found that the use of minimal restraint (e.g., placing hands on the dog’s shoulders while allowing some movement of the body and limbs) resulted in reduced dog fear, compared to full-body restraint (15). Stress-reducing practices may also contribute to fewer staff injuries among companion animal veterinarians. A North American survey study reported reduced patient-inflicted injuries rates among small animal clinics where all staff completed a stress-reducing program compared to clinics were not all or none were certified (16).

Despite the potential benefits of stress-reducing practices for dog patients, staff, and owners, implementation in practice may be challenging due to various workplace barriers. These include workloads, appointment structure, workplace culture (e.g., perceptions held by management, colleagues, or clients about stress-reducing practices), limited resources and clinic structure (e.g., costs to the clinic and clients, physical layout), and competing priorities [e.g., safety or urgency of care; (17, 18)]. Nakonechny et al. (19) report that prioritizing completion of a full examination was associated with increased likelihood of using full-body restraint, a handling technique shown to be aversive for dogs (15).

In addition to these workplace influences, situational factors, such as the dog’s behavior, characteristics, and context of the visit may also shape handling practices. For instance, handling techniques involving higher levels of restraint and certain restraint equipment, such as muzzles, are commonly reported for dogs displaying fearful or aggressive behaviors during examinations (19). Further, certain dog characteristics may also influence the veterinarian’s approach, as breed (17), sex (2, 4, 20), and age (2) have been found to influence the incidence of dog fear. Demographics and clinical experiences of veterinarians may further shape dog interactions. For instance, in a survey study, age, gender, previous clinic experiences, such as a previous dog bite, and veterinary staff role have been reported to influence the use of dog handling techniques during routine care (19).

Emerging research also suggests that veterinary professionals’ well-being may affect how they interact with patients. A common measure used to assess the professional well-being of various veterinary populations includes the professional quality of life (ProQOL) scale (21–23). This scale assesses how individuals feel in relation to helping others, including both negative (compassion fatigue) and positive (compassion satisfaction) aspects (24). Compassion fatigue is broken down into two constructs, including burnout (i.e., feeling exhausted, overwhelmed, and ineffective at work) and secondary traumatic stress (i.e., fear driven by observing work-related trauma), whereas compassion satisfaction is the pleasure or joy derived from helping others (24). Preliminary studies have examined relationships between various measures of veterinarians’ mental health, well-being, and clinical outcomes. For example, Perret et al. (22) reported that higher client satisfaction was associated with poor mental health (e.g., high perceived stress). In a qualitative interview study of veterinarians, participants reported perceived negative impacts on their interactions with clients, co-workers, and clinical care when experiencing high stress and/or poor mental health (25). Further, secondary traumatic stress has recently been found to be associated with the use of more restrictive restraints (i.e., full-body restraint) of dogs (19); however, applying restrictive handling was not detected to influence professional well-being (26). Though research is limited and mixed regarding the relationship between veterinary staff well-being and clinical care, preliminary studies and research in other sectors support the hypothesis that human well-being and behavior affect animal welfare outcomes (27–29). These insights highlight the potential for a reciprocal relationship in veterinary practice: improving patient handling and welfare may benefit practitioner well-being, and vice versa. Therefore, it is important to consider the well-being of both animals and veterinarians and explore how these factors may impact each other.

While there is growing awareness of stress-reducing strategies among companion animal practitioners, it is unknown if this translates into clinical practice. There remains a need to explore how routine examinations are conducted and what factors influence handling decisions and stress-reducing practices. Much of the existing research has focused on quantitative measures of the prevalence and risk factors for certain dog handling techniques (19), impacts of handling on dog stress (13–15), and client perceptions of care (10, 11), with relatively little qualitative exploration of veterinarians’ perspectives and decision-making processes in this context. There remains a need to gain a deeper insight into the risk factors identified in survey studies that are not limited to the researchers’ understanding; and rather, reflect the perceptions, rationales, and lived experiences of practitioners.

To address this gap, the present study employed a qualitative approach to gain insights on how veterinarians in Canada and the United States conduct routine physical examinations for dog patients. We aimed to explore the factors that influence veterinarians’ interactions with dog patients, including clinic context, patient and client factors, as well as the veterinarian well-being. Further, we also aimed to understand veterinarians’ perceptions of stress-reducing practices. This study seeks to inform strategies to better support dog welfare, the safety, and overall experiences of veterinarians during routine care. Our main research questions were: (1) how do veterinarians describe their handling approaches and responses to dogs, especially those exhibiting fearful and aggressive behavior; (2) what factors do veterinarians perceive as shaping their dog handling choices; (3) how do veterinarians perceive their well-being (i.e., burnout, secondary traumatic stress, compassion satisfaction) and emotional states to influence their interactions with dog patients; and (4) how do veterinarians perceive stress-reducing practices?

## Materials and methods

This qualitative content analysis (QCA) study was reviewed and approved by the Texas Tech University Research Ethics Board (#IRB2024-504) for research involving human participants. An inductive QCA was conducted for this study, as it is appropriate for under-researched topics, where content categories are generated from the data itself (30, 31). Individual, semi-structured interviews were used as they enable investigators to engage deeply with participants and identify meanings tied to participants’ experiences and practices concerning the phenomenon of interest (32). This study explored participants’ experiences using an interpretivist paradigm to understand meanings, experiences, and patterns that emerge from data rather than testing pre-existing theories or hypotheses (33).

### Positionality statement

Positionality involves identifying and recognizing the investigators’ potential biases that can impact a study, by describing their viewpoints and experiences (34). The first author (LN) led data collection, analyses, and manuscript writing. LN is a cis-female PhD candidate, who has focused her research on dog welfare and behavior, specifically, within veterinary contexts. LN also has previous experience working with veterinarians in various settings, including working as a veterinary assistant and conducting observational behavior research in veterinary schools. AS is a cis-female assistant professor in companion animal science with a PhD in epidemiology, having previous work experience with veterinarians and instructing veterinary students. AS research program focuses on ways to promote animal welfare through improving human-animal interactions, with an emphasis on handling, in applied settings, including veterinary clinics. KS is a cis-female associate professor in counselor education with a PhD in counseling. Her clinical and scholarly background centers on human-animal interactions in mental health and wellbeing, to include the veterinarian occupational wellness. While none of the authors are practicing veterinarians, their combined expertise in companion animal welfare and behavior (including veterinary contexts), and human social science informs the study design and interpretation. The research team approached this investigation with the following assumptions, which shaped the lens applied to the study methodology and data interpretation: the implementation of stress-reducing practices are variable across clinics and shaped by veterinarians’ personal beliefs and experiences about their effectiveness, perceived safety, and clinical context; and veterinarians value reducing dog fear but may differ in how they prioritize or operationalize this during routine examinations.

### Recruitment

Participants were recruited through purposive and convenience sampling and intended to include individuals from various regions across Canada and the United States. Participants were recruited via advertisements on social media platforms, including Facebook and Instagram. Email invitations were also sent to veterinary organizations in both eligible countries (e.g., Canadian and American Veterinary Medical Associations) to distribute to their members for further recruitment. Inclusion criteria were licensed veterinarians (≥18 years), actively practicing in Canada or the United States, and provides care to dog patients. Exclusion criteria included any known individuals of the researchers, including colleagues or friends. An *a priori* sample size of 7–19 participants was based on recommended sample sizes for recruitment of homogenous populations in qualitative research (35). Individuals who were interested in participating used the advertised link which directed them to a consent form and a brief online questionnaire. The purpose of the questionnaire was to collect participants’ demographic and practice characteristics (e.g., practice type) to (1) describe the sample and (2) inform interview probes by contextualizing participants’ clinical settings and experience. The questionnaire was created using Qualtrics survey software (Qualtrics LLC, Provo, UT, USA) and was comprised of 8 questions which collected: country of residence, gender, age, years in clinical practice, graduation year, practice type (e.g., small animal practice, shelter medicine), practice location (clinic, mobile), and stress-reducing certification (e.g., Fear Free Certified Veterinary Professional, Sophia Yin Low Stress Handling). Within the questionnaire, participants were also provided the opportunity to enter a random draw for a $100 Amazon gift card, pending their participation in an interview.

### Participants

A total of 17 participants were recruited and all participated in interviews. Participants included 15 individuals who identified as female, one as male, and one as non-binary/other. Participants resided and practiced in Canada (*n* = 4) and the United States (*n* = 13) at the time of the study and worked at a small animal practice (*n* = 13), or other practice type, such as a specialty or referral clinic, mixed animal practice (i.e., livestock and companion animal species), or shelter medicine (*n* = 5). Some participants reported having a stress-reducing certification (*n* = 7). For full details on participants’ demographic details, refer to Table 1.

**Table 1:** Demographic information of veterinarians (*n* = 17) who participated in a qualitative, semi-structured interview study.

Variable	*n* (%)
Country	
Canada	4 (23.5%)
United States	13 (76.5%)
Gender	
Female	15 (88.2%)
Male	1 (5.9%)
Non-binary/other	1 (5.9%)
Age (years)	
25–24	4 (23.5%)
35–44	4 (23.5%)
45–54	3 (17.6%)
≥55	6 (35.2%)
Graduation year	
1980–1989	3 (17.6%)
1990–1999	3 (17.6%)
2000–2009	3 (17.6%)
2010–2019	6 (35.2%)
≥2020	2 (11.8%)
Practice type	
Small animal	13 (76.5%)
Other	4 (23.5%)
Stress reducing certification	
Yes	7 (41.2%)
No	10 (58.8%)

### Interviews

Semi-structured interviews, estimated to be 60 min in length, were conducted using Zoom (Version 6.5.10) with one interviewer (LN). This software was selected for its secure video-conferencing features which enhances data security (36). Further, conducting remote interviews facilitated the recruitment of a diverse group of participants, with regards to their geographic location, veterinary experience and background (37). An interview guide was developed based on the research aim and research questions. A pilot interview with a veterinarian was held on October 25, 2024, to gather feedback and refine the questions to improve clarity (38, 39). The final guide included 11 primary open-ended questions. The number of questions included in the guide was informed by recommendations for individual interviews, where research suggests 5–10 questions additional to the first interview question, to deeply explore the research aim in homogenous samples (32). Probes were also developed for each question, and additional, non-directive questions emerged from the dialogue by the interviewer to help stimulate dialogue and gain a better understanding of participants’ responses during interviews (see Supplementary File 1 for full details). Practice details were selected as initial questions to allow participants to become comfortable in the interview setting, which were then followed by in-depth questions regarding their examination conduct for dog patients, factors influencing their patient handling, and their perceptions of stress-reducing practices.

Before recording on Zoom was initiated, the interviewer provided participants with a summary of the research aims. Participants were invited to ask questions before proceeding with the interview and were reminded that participation was voluntary. Further, they were informed that they could choose not to answer any questions and withdraw from the study until their data were anonymized; however, none withdrew their data. Informed verbal consent was provided by each participant before recording was initiated. Before the interview ended and the recording was stopped, a debriefing was provided, and participants were invited to provide any additional comments or questions. Following the interview, participants were also sent an email by the researchers that included mental health support resources for veterinarians in Canada and the United States (e.g., AVMA Wellness Page, CVMA Mental Health Resources).

Interviews (*n* = 17) were conducted between November 20, 2024, and February 5, 2025. Interview duration ranged between 14 to 70 min, and the median duration was 38 min. In addition to the recordings, notes were also taken during the interviews by LN. Interviews continued until data saturation was reached; this occurred at 17 interviews, after which additional interviews failed to contribute new insights to the emerging categories (30, 35). Audio recordings were transcribed by Zoom and were then reviewed by LN in Microsoft Word 2011 (Microsoft Corporation, Redmond, WA, USA) for accuracy. Any personally identifying information in the transcripts was removed to ensure participants’ anonymity, and then recordings were permanently deleted prior to formal analyses.

### Data analysis

Demographic data were analyzed in Microsoft Excel 2011 (Microsoft Corporation, Redmond, WA, USA). Interview transcripts were imported and analyzed in NVivo (Version 14.24.3, QRS International, Melbourne, Australia), a software program for qualitative data coding and analysis. Transcripts were analyzed using QCA, as this approach is particularly useful when research on a topic is limited and the goal is to describe and understand the phenomenon (30, 40). All transcripts were reviewed by LN for data familiarization and to explore initial patterns. LN open-coded five transcripts to develop an initial codebook using a combination of descriptive, *in vivo*, and process coding techniques. Coding consisted of an iterative process in which transcripts were first open coded by identifying significant statements (i.e., meaning units), generating unique codes for each statement, and memoing researcher thoughts and ideas on emerging concepts. After initial coding, codes were reviewed for redundancy and then sorted and grouped into similar ideas to formulate the sub-categories and categories. Throughout this process, codes were generated inductively, or emerged from the raw data rather than from pre-established codes or frameworks (41). To enhance credibility and trustworthiness of the data, peer debriefings with AS were held regularly throughout the analytic process to discuss emerging codes and categories. Following discussions with AS, LN modified the codebook by comparing, adding, merging, and/or splitting codes to reduce redundancy and enhance refinement. Through iterative coding and research memoing, a final codebook was produced when no new codes emerged from the data. Subcategories and main categories were abstracted by LN and AS and finalized when consensus between both investigators was reached. An audit trail was maintained by LN throughout the analysis to enhance dependability by documenting changes and rationales to the codebook. KS provided an external audit to assess analytic rigor and interpretive consistency. Unique identifiers (e.g., P1) were assigned to participant quotes and square brackets (i.e., […]) are used to indicate when a quote was shortened or a word was modified to provide context. For example, ‘they’ was changed to ‘owners’ or ‘patients’ in some quotes to provide the reader with better clarity. Verbatim quotes were included to provide context and highlight important findings for the categories (See Supplementary File 2).

## Results

### Overview of categories

This study explored how veterinarians perceive and navigate their interactions with dog patients during clinical examinations. In particular, we explored: (1) how veterinarians describe their handling approaches and responses to dogs (particularly those exhibiting fear or aggression), (2) what factors veterinarians believe shape their handling practices, (3) how veterinarians’ own well-being and emotional states influence their interactions with patients, and (4) veterinarians’ perceptions of using stress-reducing handling practices (including perceived benefits and challenges). Analysis of the interview transcripts yielded four major categories and 11 sub-categories corresponding to these questions (see Supplementary File 2). Each category and subcategory are described in detail below, supported by exemplar quotations from participants.

### Category 1: approaches to dog handling

Approaches to dog handling refer to the strategies that participants used to handle dog patients during examinations, particularly those presenting with fearful or aggressive behavior. Participants described how they help dogs stay calm using stress-reducing practices, and what forms of restraint and handling tools they use to facilitate clinical assessments and safety. They also reported adapting their examination conduct and procedures, including the personnel (e.g., technicians) involved with handling. This category had three sub-categories: stress-reducing practices (8 codes, 23 sub-codes); restraint and handling tools (2 codes, 9 sub-codes); examination conduct and procedures (3 codes, 10 sub-codes).

#### Subcategory 1.1: stress-reducing practices

Stress-reducing practices encompassed intentional, non-forceful strategies used to reduce dog fear and aggression and to promote calm behavior during visits. Participants described considerations for visual, auditory, olfactory, and tactile stimuli in clinics and modifications they employ to reduce stress for dog patients. This included minimizing conversation, speaking quietly during appointments and avoiding loud noises and sudden stimuli, such as knocking on the examination door. P7 shared: *“I leave the door open because some dogs are reactive when you open the door…so that we can go into the exam room without the animal getting any more agitated that they have to.”* P2 noted calmer behavior after reducing foot traffic in the waiting room: *“…the dogs and cats are so much happier because there’s no drama in the waiting room. There’s nobody walking in. There are no doors opening, there’s no talking, there’s no barking.”* Several participants described using environmental modifications such as calming music, pheromone diffusers or sprays, and non-slip surfaces on examination tables to reduce dog stress. Others also dimmed lights, used visual barriers or kennel covers, and incorporated home-like furnishings in examination rooms to help create a more comfortable environment.

All participants (17/17) described the use of treats, play, petting, and/or verbal praise. Treats (such as whipped cream, squeeze cheese, dog treats) were used to reduce fear, facilitate cooperation (e.g., used as distractions), and create positive experiences. Several participants provided treats to dogs upon clinic entry, throughout the examination, and after the appointment: “…*usually when I walk in, the first thing I’ll do is say, hi, to the owner from afar, and then I usually will crouch down on the ground, and then just throw treats on the floor, just to get the pet like used to the fact that I’m in the room*” (P6). Others used ‘licki-mats’ covered in squeeze cheese, peanut butter, or whipped cream to keep the dog occupied as they conducted an examination or palpated certain areas of the dog’s body.

Besides treats, some participants also offered toys to engage dogs in play, petted dogs, and used positive verbal praise. Several mentioned using ‘happy visits’ to build trust with dogs, where dogs would attend the clinic to only receive positive experiences (e.g., treats, pets, playing with staff) and no examination or procedures were performed. These visits were described as successful in reducing dog reactivity or aggression over time (e.g., 4 to 6 visits). P7 shared: *“…[the dogs] come in once a week and [we] just give them treats and let them play and have a good visit.”* Participants also adapted their handling strategies based on the dog’s behavior, perceived stress, and previous experience. This included decisions about where and how the examination takes place, the examination sequence and pace, and handling techniques used. Many participants (12/17) allowed dogs time to acclimate before beginning a physical examination. For example, they allowed dogs to freely explore the room and avoided physical interactions or directly approaching visibly fearful dogs. P14 shared that they have the option to examine fearful dogs in a larger clinic room:

*‘‘[We] have a larger room…it’s twice the length of our normal exam room. So, I always go in there to allow the dog who is nervous to physically have more distance from me…and then we have more time to make a rapport with the owner and to discuss a handling routine.’’*

Participants described acclimation as an opportunity to visually assess the dog’s body language and observe any potential health issues (e.g., gait abnormalities, signs of pain). Most participants discussed using a ‘less is more’ or non-invasive approach with physical restraint where *“…the less restraint we do with them, the better”* (P2). Several also mentioned applying ‘touch gradients’ where pressure applied during body or limb palpations is gradually increased, and the dog is examined initially in more comfortable areas (e.g., body), while working towards less comfortable areas (e.g., paws, ears).

#### Subcategory 1.2: restraint techniques and tools

Restraint techniques and tools referred to equipment- or technique-based strategies used to maintain safety or facilitate clinical procedures. Generally, minimal restraint was initially used, such as holding the dog’s leash or collar, or passively holding the dog: *“I like to generally just hold the dog, not necessarily restrain them, but you just kind of loosely keep your arms on them.”* (P7). Several participants described starting an examination with a standing restraint, where *“one arm is around the neck, and one other arm under the abdomen”* (P4).

Participants emphasized the importance of reading subtle behavioral cues to decide when to pause or stop an examination, or use chemical restraint. P10 described a decision-making process when encountering a stressed patient:

*‘‘…if the dog is obviously fearful, we'll take a step back and assess. You know. What can we get done visually? Do they need to come back with sedatives? Schedule another visit? If it's an emergency, do we need to use injectable or oral sedation during the visit to reduce stress?’’*

Many participants (15/17) described opting for chemical restraint when dogs showed higher levels of fear, aggression, or were anticipated to be fearful and/or aggressive. For example, injectable or oral sedation (e.g., acepromazine, dexmedetomidine) was administered during appointments, or examinations were stopped and rescheduled with pre-visit medications, such as gabapentinoids (e.g., gabapentin) or serotonergic agents (e.g., trazadone). Some participants avoided using chemical restraint if dogs showed fear, but not aggression, during days with high caseloads. For example, P13 said:

*‘‘I feel more inclined [to] push through a fearful dog. And the reasons for that is… there is a time constraint…if the dog is cooperative and just freezing, I'm still going to do my examination. But if there's any outward aggression for staff safety, we knock them down.’’*

P1 said that using chemical restraint in certain situations could detract from an accurate diagnosis, as the dog’s *“pain or gait is muted.”* Chemical restraint was also described as being important for preventing dog injuries. For example, P11 explained that fearful dogs may injure themselves (e.g., cruciate ligament injuries) while struggling against physical restraint.

Signs of fear or aggression (e.g., head whipping, growling, whale eye) prompted some to employ tools such as towels, blankets, e-collars, or muzzles. For example, P15 described: “…*I usually am reaching for a muzzle, especially if they show warning signs of aggression, so like snarling or growling, or snapping that kind of thing.”* Towels or blankets were used to reduce the dog’s movement or prevent a bite, by wrapping the dog’s body or head; this was also more often applied to small dogs (vs. larger dogs). In terms of muzzles, participants expressed diverging preferences for basket and cloth muzzles. Those who favored cloth muzzles mentioned it was primarily for their ease of application. However, more preferred basket muzzles because they allowed dogs to pant, eat treats, breathe more comfortably, prevent nipping, and permitted an oral examination. For example, P14 explained: *“…you can be nipped by a dog with a cloth muzzle. I just think the basket muzzle with the total coverage is more reliable.”* Several preferred that dog owners apply their own muzzles to dogs, as this seemed to evoke less aggression (vs. having it applied by the veterinarian). Alternatively, some preferred that an experienced technician apply a muzzle to reduce safety risks: *“…techs are generally a lot better at getting a muzzle on a resistant dog than I would be. So, I let them take a shot. And ultimately, if everything proves impossible, then that’s a chemical restraint situation”* (P14).

#### Subcategory 1.3: examination conduct and procedures

Participants described their typical examination conduct, including the sequence, location, and structure of physical examinations in response to dog behavior, clinical needs, and contextual constraints. Participants described selecting examination location based on dog size, behavior, and comfort, with many preferring floor-level examinations for larger dogs, and a table for small dogs. Several participants explained that having a dog on a table aided control and ease of handling: *“…they’re a little bit nervous up there…but it grounds them a little bit, versus being on the ground where they’re hyperactive and wiggling and they won’t hold still”* (P13). Others trialed the table with small dogs but switched if the dog appeared uneasy: *“If it’s a smaller dog that could go on the table… we’ll give it a try. And if the dog seems uneasy, I just put them back on the floor”* (P14). A few participants also described having physical limitations that necessitated their use of tables to examine most patients, regardless of the dog’s size.

Bringing the dog to other locations in the clinic (e.g., treatment area) was preferred by some participants when certain procedures needed to be performed (e.g., bloodwork), or if the dog was perceived to be difficult to handle. For example, P3 explained: “…*if the dog’s given us a particularly hard time…I just take the leash and walk down the hallway…and we can get what we need to get done back in the treatment area.”* Dogs that were described as *“too frantic to accomplish anything”* (P11), or *“known biters or actively trying to bite”* (P4) were reasons that resulted in dogs being examined in the treatment area. Others performed curbside examinations for dogs unable or unwilling to enter the clinic: *“We prefer to work inside the vet clinic, but when the time is appropriate, we do work outside in the parking lot…there are some of those dogs who are so terrified, they won’t come in”* (P3). Examination location was generally a flexible, situation-dependent choice aimed at reducing stress while preserving control and safety for patients, clients, and staff.

Participants described using systematic yet flexible examination procedures, often beginning with a ‘head-to-tail’ approach that was adapted based on each dog’s comfort, behavior, and clinical needs. As P13 explained, *“I have a very methodical approach from starting from nose and work my way all the way to the tail… But if I have a dog that’s super fearful or say they’re painful…I will absolutely skip that [area], and we do that very, very last to stress out the dog less.”* This modification was common, where completing more invasive or uncomfortable procedures, such as rectal temperature checks, nail trims, or anal gland expression, was delayed until the end of the examination.

Patient pre-screening was used to anticipate handling challenges before the appointment. For example, reception staff were trained to identify ‘red flags’ during scheduling and communicate them to veterinarians, such as dogs with a previous history of aggression at veterinary clinics. This proactive triaging process allowed veterinarians to prescribe pre-visit medications and prepare appropriate handling plans. Documentation also played a role in promoting continuity of care and preventing repeated stressors across visits. Participants emphasized maintaining detailed behavioral notes in medical records to guide future handling. P4 explained, *“…usually, if there’s a particular way that seems to make the dog more comfortable or less aggressive, then we make a note of that for sure in the chart, and we do it that way every single time.”* Others described using standardized fields in their electronic medical record systems to record specific handling preferences, triggers, or successful techniques.

Participants described varied preferences for an owner’s level of involvement with handling during examinations. Several reported routinely engaging owners, such as providing distractions for their dog (e.g., feeding treats, petting), or following veterinarian-guided restraint: *“I instruct them in how to hold the dog, that I can have good access to listen to the heart and look at the eyes and ears and mouth”* (P14). Several participants also described limits and risks to owner involvement. Some preferred to minimize owner handling due to bite risks and liabilities: *“I don’t want the owners to get involved and possibly get bitten”* (P1). Others mentioned that owners may hinder proper examination of the dog, requiring redirection or technician assistance: *“Most owners are not very useful when it comes to helping with any dog handling… the dog is wandering this way and that way”* (P12). Overall, owner involvement was described as contingent on safety, the dog’s behavior, and the owner’s ability to follow instruction.

Many participants (12/17) described assistant or technician-led intake and support throughout the visit, including early history-taking and signaling anticipated difficulty: *“We will usually come in after the technician or assistant has taken the history…they go in first on their own…and if [there is] something really obvious, like this is going to be difficult, they’ll let me know so we can kind of brainstorm what best approach to take”* (P10). Technicians commonly performed restraint during examinations and several provided guidance to adjust technicians’ handling:

*“…technicians are often trained to restrain sort of maximally… I will tell my tech…I think [for] this one, less is more…and I’ll just sort of try to make sure they are positioned so that they can grab the head if they need to…or have a hand on the collar or the leash”* (P11)

P13 described having structured team roles (e.g., a scribe and a designated handler) to maintain safety and efficiency of the appointment. A few participants described working alone, mostly based on personal preference. For example, P7 said: *“…depending on who is helping me, I get a little nervous. I like to restrain [dogs] without them.”*

### Category 2: factors affecting dog handling

Participants described various factors that influenced their handling decisions, including dog patients (e.g., the dog’s behavior, health, and physical characteristics), the dog owner (e.g., presence, expectations and preferences), the workplace context (e.g., clinic policies, resources, and veterinary team), and their previous training (including veterinary school) and experiences. This category had four sub-categories: dog patients (3 codes; 5 sub-codes); dog owners (3 codes; 5 sub-codes); workplace (4 codes; 2 sub-codes); formative experiences and training (4 codes; 7 sub-codes).

#### Subcategory 2.1: dog patients

Participants described dog behavior, specifically levels of fear and aggression as primary determinants of how they approached handling. Many participants (15/17) said that they focus on the identification of dog stress signals. For example, P9 explained:

*‘‘…a patient who's either afraid, anxious, or fearful, or a combination of those…that immediately changes how we see them… we have to figure out what is going to make this patient feel more comfortable in this context.’’*

Some participants used formal fear scales (e.g., Fear Anxiety Stress or FAS scale) to assess fear and determine appropriate responses: “…*we’ll evaluate the FAS score… if there’s any sign of like biting or being too tense then we again we back off and reevaluate”* (P10). Several participants identifying certain body language or behaviors (e.g., dog posture, aggression) as thresholds for stopping an examination and rescheduling with pre-visit medications. For example:

“…*if the animal is showing defensive postures, or even worse, offensive postures…I'll just say…we got to stop and rethink this. And we're either gonna reschedule that dog to come in on some sort of, you know, pre-visit pharmaceuticals.”* (P8).

Dog health status and certain characteristics, such as the dog’s size, age, and breed also were discussed as influential. Dog size commonly guided examination location, where many preferred floor-level examinations for larger dogs while reserving the table for very small patients: *“If it’s a small dog… then I’ll put it on the table.”* (P11). Dog age and mobility also mattered; P5 explained: *“…if they’re an older dog, and have trouble getting around… I’ll have them on the ground.”* Some positioned small dogs where they felt most secure, including the owner’s lap: *“We’ll often examine little dogs…on their owners’ laps …, because they can feel more secure that way”* (P10). Breed was occasionally noted as shaping expectations for handling, particularly in relation to past experiences. For example, P11 observed, *“…three of the most aggressive dogs I think I’ve ever worked on were Rottweilers… all required injectable sedation.”* Health status further shaped the order of the exam and pharmacologic choices, with veterinarians deliberately deferring examination of known or suspected painful body areas until the end of the examination to minimize dog distress.

#### Subcategory 2.2: dog owners

Veterinarians’ handling approaches were influenced by their interactions with owners, whose presence, expectations and preferences, and involvement during examinations was perceived to be beneficial or create challenges. Some participants explained that certain dogs showed aggression when their owner was present during an examination. In such cases, they would opt to separate the dog and owner. For example, P11 explained:

‘‘…*there's a fair number of dogs that are owner defensive or protective. In which case, obviously I would rather them be away from the owner…there's a good number of dogs that are perfectly reasonable and handleable…with the owner not present. But with the owner present, they are barking uncontrollably…and [are] very aggressive, only in the presence of the owner.’’*

Several participants also mentioned requesting consent from owners when using certain restraint tools: *“If we have to place a muzzle, I’ll run it by the owner first”* (P5). Several also described paying attention to body language and facial expressions of owners during examinations and modifying their restraint accordingly. For example, P5 said:

*‘‘If I'm seeing any signs that [the owner is] not [comfortable with the level of restraint], …, I'll kind of back up a little bit, and I'll say, I know this is stressful on fluffy…but we kind of need to get this done…’’*

Likewise, P14 discussed how dog stress can affect owners: *“[owners] love their dog, and they don’t want to see them be scared.”* P1 explained that some owners feel embarrassed about their dogs’ fearful or aggressive behavior: *“…[owners] are embarrassed. They’re afraid for me. I’ve had many, many people tell me, just don’t get bitten… I think there’s embarrassment and fear that I’m going to get hurt.”*

Some participants described having frequent communication with owners during the examination to help support their handling choices. For example, some would verbally describe their handling to owners in a conversational manner to support mutual understanding. P1 explains: *“I make sure that families know that I know what they are thinking, and what the pet is thinking, and that I’m taking that into account in my handling.”* Several participants also described navigating conflicts between owners’ preferences, expectations, and their own handling methods. For example, some felt that owners equated physical restraint with effectiveness: *“[Owners] don’t feel like a physical exam is complete without hands on”* (P1), which conflicted with participants’ preferences to visually assess the dog as much as possible. P10 described: *“…we have actually had some clients leave the practice because they think we’re too like soft on their dogs. And to me, that’s just not a good fit.”* P14 explained a similar challenge, where owner preferences or expectations conflicted with their professional values:

*‘‘I certainly could get two or three techs and just overpower this dog and do [the examination] against his will. I don't. I'm not willing to do that…I think it's a huge disservice to the dog. Not only [is it] sort of against our oath to be, you know, to be helpful and not cause harm to animals, but also for my future with that patient. That dog is going to be terrified of coming here forever, and anytime he needs more urgent care…and it's better for everyone to avoid those situations. But for the owner, all they're hearing was like, I have to come back here another time. I have to pay a pile of money, and it's just, you know, the suspicion that the greedy vet is like gouging you and making you do things just to get money.’’*

Others expressed feeling stressed about owners’ reactions to their advice, such as rescheduling examinations with pre-visit medications for fearful dogs. For example, P7 said: *“I get nervous because I don’t know if they’ll be on board with coming back…are they gonna not be happy about doing that?”* Several also explained that a lack of owner compliance with administering pre-visit medications created issues. For example, owners forgetting to give their dog pre-visit medications resulted in the dog being fearful for the examination, and this was described as frustrating by some participants.

#### Subcategory 2.3: workplace

Participants described how their work environments shaped their handling choices. Across participants, the workplace emerged as a context where individual and clinic priorities intersected, specifically with applying stress-reducing practices. Participants who owned their own practice or worked at clinics where management permitted flexibility with appointment length and decisions about when to stop and reschedule examinations or procedures were conducive to stress-reducing practices. P2 explained: *“…we’re lucky that I’m a one-vet practice because we can kind of do what I want… we have half-hour exams… we’re not like, just, you know, doing everything as fast as we can to get it all done.”* P1 expressed competing demands between using stress-reducing practices and being efficient: “…*in the name of efficiency, low stress is kind of pushed aside at times…we think if we’re being efficient, we grab that pet, poke them with a needle, and send them on their way.*” P1 also reframed efficiency as compatible with dog welfare, explaining that “…*the better you are at low-stress, the more efficient you can actually be.”* Conversely, P10 described how some stress-reducing techniques, such as providing treats, was perceived as infeasible during high workloads: *“…if we’re like, really behind, and I’m busy, and I really just want to get this done…then I don’t have time to, you know, sit on the floor and feed one treat, and then two treats and three treats.”*

Staff training also shaped participants’ handling decisions. Those with support staff trained in stress-reducing practices described feeling empowered to safely manage fearful or aggressive dogs and felt they were at lower risk for injury, compared to clinics where these practices were not used P10 explained:

*‘‘…in my early days it really freaked me out, especially the clinics that I was working at before were not Fear Free. I did not have these tools. There was much more of a risk of getting injured. But now I feel safe. And the risk is never zero of an injury to myself, staff or owners, but I think it's dramatically lower…and it's nice to work with staff that's supportive of that, too, because I know that's not always possible.’’*

Participants also noted that support staff with limited handling skills or experiences heightened safety risks:

*“I would never hand a green technician a muzzle and say, here, go muzzle that dog. That’s a recipe for somebody getting bitten…if my handler is not in a space where they are comfortable with restraining and keeping everybody safe, then it's not a good situation”* (P17).

Participants expressed having confidence when *“staff are trained the same way”* (P15) and *“all on the same page”* (P2).

#### Subcategory 2.4: formative experiences and training

Participants attributed their handling approaches to a combination of their veterinary education, early work experiences (e.g., internships), professional development (e.g., conferences, workshops) and certain experiences, such as the COVID-19 pandemic. While veterinary school provided the foundation for technical and clinical knowledge, participants described widely variable exposure to stress-reducing practices and patient-centered care. These gaps were often bridged later through mentorship and continuing education, which most participants credited as more influential than veterinary school.

Participants’ recollections of veterinary school ranged from highly limited instruction in animal behavior and restraint to more patient-oriented programs. Several participants described procedure-focused teaching that prioritized efficiency and safety over the patient’s experience. P1 reflected:

*‘‘I don’t recall chemical restraint for cats and dogs being used a whole lot…more often than not, you’d see [dogs] squished into a corner, covered with a towel, something fairly fast and scary, and then getting the shot that hurts.’’*

Similarly, another said, *“We were definitely taught that you had to restrain animals properly so that you could get the job done…it was about making sure no one got bitten, not about how the patient felt”* (P9). Others highlighted the near absence of instruction about handling fearful dogs: *“I can’t recall any specific handling of fearful dogs being reviewed, other than how to get a muzzle on without getting bit”* (P10). Few participants (3/17) described receiving exposure to stress-reducing practices, usually linked to specific mentors or institutional emphasis. For example, P6 shared, *“…we used minimal restraint, baby food, whipped cream, anything to make it a positive experience. If it was too overwhelming, we would stop and send them home or sedate them. That was really heavily taught at school,”* and P14 reflected: *“We had an [instructor] who was very committed to animal welfare and behavior.”* Across participants, veterinary school was described as valuable for clinical and theoretical knowledge but generally insufficient for preparing them to confidently manage fearful or aggressive patients safely and in a lower stress manner.

Experiences after graduating from veterinary school such as mentorship, continuing education (CE), and working at various clinics primarily shaped participants’ current handling practices. For some, working at clinics with skilled veterinarians, technicians, dog trainers and behaviorists were described as pivotal: *“All of my dog and cat handling experience and tools have come from practice…I learned everything from the technicians I worked with”* (P11). Another explained, *“I worked in shelters that offered CE for professionals, bringing in trainers like Karen Pryor…that’s what really shaped my idea of animal interaction”* (P12). Some also discussed development of a cautious approach with dogs, based on previous negative experiences (e.g., bitten by a dog). Personal experiences with dogs outside of clinical work also played a formative role, including ownership of dogs with behavioral issues. For example, P2 states: *“One of my dogs is reactive, a fearful Malinois…he’s been my learning curve.”*

The COVID-19 pandemic further reshaped how participants approached patient interactions. Many described how the disruption to normal operations prompted creative adaptations that unexpectedly improved dog welfare. P2 shared:

“*During COVID, we knew it was stressful, so we literally sat in the room and played with the dogs. We had toys and treats, and we just played with them. It went a long way to building relationships with the dogs”*

Others found that outdoor or curbside examinations reduced dog fear*: “We realized that we could do stuff outside… if we had to bring [the dog] inside, we’d have to use sedatives”* (P2). Reflecting on the period, another participant described the pandemic as, *“…a great learning tool…it made us think differently about where and how we examine dogs to keep everyone calm and safe”* (P8). These adaptations demonstrated how necessity and environmental change could expand practitioners’ repertoire and application of stress-reducing strategies and reinforce the importance of flexibility and creativity in clinical care.

### Category 3: veterinarian professional wellbeing

Participants described a balance between the emotional demands of practice and the fulfillment derived from meaningful work. Compassion fatigue reflected the draining effects of moral distress, clinical work, and repeated exposure to animal and owner suffering, while compassion satisfaction restored motivation through positive outcomes, connection, and purpose. This category had two sub-categories: compassion fatigue (3 codes; 19 sub-codes); compassion satisfaction (2 codes; 4 sub-codes).

#### Subcategory 3.1: compassion fatigue

Participants described compassion fatigue as arising from the stress and emotional intensity of clinical work, challenges with patient care, and the cumulative pressures of long hours and client expectations. Almost all participants (15/17) described experiencing burnout at some point during their career, as well as secondary traumatic stress (14/17). Repeated euthanasia, particularly when it was the result of owner finances, was described as a draining experience in practice. *“It was a lot of euthanasia… for financial constraints versus the pet really needing to be put to sleep. It was exhausting and tiring,”* P2 explained, adding, *“…in one weekend we did 21 in one shift… it really tears your heart out.”* High-volume or under-resourced work, where long hours compounded fatigue was described by several. For example, P11 reflected: *“I was working in a very high-volume practice with on-call 24 hours, 50 to 80 hours a week… that was my first job, and that’s when I would have considered myself burnt out”* (P11). Those working in emergency medicine described even greater strain due to high caseloads and frequent euthanasia. Others recounted experiencing distress when performing procedures that conflicted with their values, or when owners’ financial constraints resulted in euthanasia: *“Being asked to do things that you felt were wrong, doing nail trims or vaccines on terrified animals, or euthanizing healthy dogs, was incredibly stressful”* (P9). P6 described a behavioral euthanasia where the owners’ distress was traumatic to observe: *“The owner was screaming, sobbing… I had to leave and cry because I was so traumatized. I have never been so traumatized witnessing someone else lose it.”* Difficult client interactions were described by several participants as fatiguing and stressful, particularly when communication broke down or they disagreed on clinical procedures or handling approaches.

Participants described a range of impacts stemming from compassion fatigue, often reflected in less patience, reduced empathy, and feelings of ineffectiveness during clinical work. Many recognized that exhaustion compromised the quality of care they were able to deliver. P11 acknowledged, *“Yeah, you care less,”* while P14 reflected, *“If you approach an appointment when you feel tired and impatient, the patient and the client cannot be getting compassionate, good service from a practitioner who’s not doing well themselves.”* Several participants described ‘pushing through’ examinations and recognizing the limitations of their focus or energy. As one explained, *“… I’m more inclined to just crank this out, versus another day when I’d take more time or adjust medication”* (P13). Another explained that they were less likely to apply stress-reducing practices: “…*if you have more burnout, or you’re just not happy with your environment, it’s less likely that you’re going to want to do low stress or educate people”* (P6). Feelings of self-criticism accompanied these compromises, as described by P17:

*“… I looked at myself as inadequate…I felt like I wasn’t able to provide their owners with the amount of time and information, and patience and care that I wanted to give them…it just felt hurried to me… So, it felt like I wasn’t giving my all…it was this constant feeling…I wish I could do more, but I don’t have time to do more…because I’m being stretched thin.”*

Several felt that experiencing compassion fatigue did not impact their interactions with animal patients; rather, it negatively affected their interactions with colleagues and clients, describing less patience or being blunt. P9 explains: *“If you’re not feeling your best, it’s easy not to be the best version of yourself… your filter changes with people when you’re burned out.”*

Participants described various coping strategies for compassion fatigue, ranging from short-term emotional regulation and compartmentalization to boundary-setting and lifestyle changes. In the moment, many described doing a ‘mental reset’ between cases: *“I can compartmentalize… take a deep breath, close your eyes, count to 3, and then go to your next appointment”* (P16). Another participant intentionally shifted their focus: “…*there is probably more sadness and frustration with this profession than we’d care to admit. And I don’t wanna focus on that. And that’s a deliberate choice*” (P3). Many emphasized seeking peer support, acknowledging their emotions, and deliberately structuring work to protect recovery (e.g., limiting caseloads, scheduling decompression time, taking lunch away from the clinic, avoiding triggering procedures). Several described that cutting back hours was an effective strategy for managing compassion fatigue. Many also described benefiting from connecting with colleagues, for example:

*“…talking it out with other people and relating those experiences to other veterinarians and other animal healthcare people can be really good. Because there's a lot of support…everyone's probably dealt with very similar things in the course of their career…it feels so good to have my colleagues come and say, hey, you know I heard that happened. Sorry to hear that…you get that community feeling”* (P16)

#### Subcategory 3.2: compassion satisfaction

All participants (17/17) described deriving compassion satisfaction from their work, from experiences such as meaningful interactions with patients and clients, successful clinical outcomes, and a sense of professional purpose. Participants described getting compassion satisfaction from moments of connection, purpose, and visible impact, when clinical work aligned with their values and produced meaningful outcomes for animals, owners, or colleagues. Many drew fulfillment from collaborative relationships with clients who were engaged and appreciative, as P10 noted: *“Just really making a good connection with [owners], knowing that they trust me, and I know that they really care about their pet. I think those two things are what make it good.”* Successful treatment outcomes and clear communication were described as reinforcing motivation and professional confidence: *“When we’re all on the same page with the case… [the owner] actually listened to me, and we had a plan. That’s when it feels good”* (P7). Happy moments, such as wellness visits, working with puppies, and positive follow-ups, also helped sustain emotional balance: *“I see lots of fun appointments too…you have to allow that balance to exist in your brain and in your emotional state”* (P11).

Tangible improvements in animals’ health or behavior and helping owners provided satisfaction: *“It’s like the most satisfying thing in the world… you’ve actually helped that patient out, the client’s happy, and it makes things a little bit worthwhile”* (P16). P3 reflected that clinical ‘wins,’ however small, provided happiness and optimism amid emotionally taxing work*: “We start every staff meeting with celebrations… we cannot do the sad work that we do over and over without grabbing hold of those glimmers.”* Compassion satisfaction also shaped participants’ demeanor during appointments, promoting patience that carried into client and team interactions. As P16 explained, *“If I’m in a good mood…it can be a lot easier to just take things in stride and approach challenges calmly.”*

### Category 4: perceptions of stress-reducing practices for patients

Participants’ perceptions of stress-reducing practices reflected both endorsement of its’ benefits and recognition of practical challenges. Many described these approaches as improving experiences for the patient, owner, and veterinary staff, while fostering a more enjoyable workplace. However, several participants discussed that limited resources, time constraints, and inconsistent team support often restricted their use, leading participants to selectively apply stress-reducing methods or perceive them as infeasible in certain situations. This category had two sub-categories: perceived benefits (3 codes; 11 sub-codes); perceived challenges (2 codes; 7 sub-codes).

#### Subcategory 4.1: perceived benefits

Some participants associated the use of stress-reducing techniques with enhancing both animal welfare and human well-being. P9 explained, *“The biggest benefits of low-stress handling are the emotional and physical welfare of everybody… the staff, yourself, the pet parents, and definitely the patients.”* Participants described how these methods helped build stronger veterinarian-client relationships and trust through cooperative care, where restraint is minimized and not forced.

*“It can really improve the relationships that you have with the clients, because they feel like their pet is being handled in a humane way. Therefore, you can do better care for the animal, because the client trusts you”* (P10).

Stress-reducing practices were also credited with improving ease of handling and safety for both patients and staff. Participants described less fearful and more cooperative dogs. P2 reflected: *“We’re kind of working with them instead of forcing them…it builds a better relationship with the dog, so they’re not as scared coming in the next time.”* Others emphasized that these methods enabled more thorough or productive examinations: *“Gaining the patient’s trust is paramount to doing difficult things… if we can do it in a low-stress manner, we can accomplish more”* (P3).

Participants also discussed benefits to clinics and team well-being. Participants stated that stress-reducing practices reduced challenges with dog handling. For example, P6 explained, *“I’ve worked at clinics that don’t do low stress, and my clinic where I do low stress, it’s night and day… everyone’s struggling, over-restraining, dogs are panicking.”* Others noted that they encounter misperceptions from other team members about stress-reducing practices, particularly that it reduces clinic efficiency. P6 felt efficiency (in terms of number of appointments) did not differ between clinics who do and do not practice stress-reducing techniques “…*if you have people trained and comfortable, you can do low-stress, get in and out, and see the same amount of appointments as somebody else not sedating any dogs.”*

#### Subcategory 4.2: perceived challenges

Several barriers prevented the implementation of stress-reducing practices, despite widespread agreement on its value by all participants (17/17). Challenges stemmed from limited staffing, time pressures, clinic space, and costs, as well as lack of colleague and owner support or understanding of these approaches. For example, P16 explained:

*‘‘…I think one of the big challenges is trying to convince owners the value. Some owners–not all of them–but some owners, don't really see the value of the low stress handling… Sometimes when they see it, they're like, oh, I get it now. I see why you're talking about that…Sometimes they're more just like, just get it over with, get it done, and they don't care so much about what is going on necessarily. Or they don't* seem *to. Trying to emphasize and to reach the owners that way can be a little challenging sometimes.’’*

Several emphasized the need to balance ideal practices with operational constraints, acknowledging that while stress-reducing practices were preferred, it was not always feasible within high caseloads, limited resources, or fast-paced clinical environments.

Several participants described the use of stress-reducing practices as highly dependent on management and interpersonal support. Even when participants were personally committed to stress-reducing practices, they encountered lack of ‘buy-in’ or support from colleagues: *“…the limitation that stands out to me is just getting uptake from other colleagues and staff members”* (P14). Further, several mentioned a lack of education among colleagues about stress-reducing practices: *“…people don’t have enough information a lot of the time, to know how easy it is to make those changes that can make such a big difference”* (P9). Several also commented that having sufficient staff available and trained in stress-reducing practices is essential to employ this type of approach. Beyond perceptions, participants described limited time, space, and costs as constraints. Short appointment times and rigid scheduling structures left little opportunity for dog acclimation, adjusting handling to the dog’s behavior, or discussions with the owner. As P7 noted, *“We as a profession have really pushed ourselves to just get stuff done… if you’re in a 15-min time slot…you’re going to feel really awkward to try and stop and take 30 min to sit with this pet”* (P7). Others experiencing practical obstacles such as a shortage of quiet examination rooms or areas large enough to permit fearful dogs’ adequate space to retreat. P10 discussed time and costs associated with sedation and pre-visit medications or ‘chill-packs’:

*‘‘…it does take more time, and it can be frustrating for us and for people to have to come back on like a chill pack. They have to spend more money. Sedation injectable is not cheap. So financially, it can be a burden for people, and just a big time suck.’’*

Some also explained that costs associated with becoming certified in or accessing stress-reducing education was prohibitive: *“I kind of hate that you can’t just go and read it. You have to pay money and sort of subscribe to it”* (P12). Another recurring challenge involved balancing dog stress with clinical needs. Some participants described making case-by-case judgments or limits about when stress-reducing practices could be implemented and when completing the examination or procedure took precedence. P11 explained: *“You’re always weighing the benefits of getting the thing done with the stress you induce doing it…sometimes it’s worth the stress, and you fight through; sometimes it’s not, and you reschedule or sedate.”* Likewise, P13 explained: *“I feel empathy for the animals, but there’s a limit, I still need to do my job.”* Others discussed using certain aspects of stress-reducing practices when perceived to be impractical or “not effective for every dog”. P8 explained:

*‘‘I pull bits and pieces. I think the general tenets of it are marvelous. That doesn't necessarily mean I'm agreeing with all of the details. Though I think you have to pull from that what you find to be useful and functional’’*

P4 held a similar perception*: “…it’s a little bit more time consuming, I think, especially on like a really busy day. You know, just trying to take the time to be able to do that with the patient…and I do think there’s some [dogs] that just it just doesn’t work for.”*

## Discussion

This study provides insights into veterinarian handling decisions, their experiences of compassion fatigue and satisfaction, and the perceived benefits and challenges of stress-reducing practices. Findings demonstrate dynamic handling choices that are weighted against patient behavior, perceived safety risks, urgency of clinical care and diagnostic accuracy, owner perceptions and expectations, clinic factors (e.g., staffing, resources), and professional well-being.

Participants commonly described using strategies that align with recommended stress-reducing practices. For instance, this included reducing environmental stressors (e.g., using non-slip surfaces, visual barriers, minimizing noise), providing positive experiences (e.g., provision of treats and toys), starting with minimal levels of restraint, and administering pre-visit or in-clinic anxiolysis/sedation for fearful or aggressive dogs (6, 12). Participants emphasized observing early stress signals, and pausing, stopping or rescheduling examinations to prevent which further fear aligns with recommendations to prevent negative veterinary experiences that can condition and intensify fear dog fear during future visits (2, 9). Dog behavior upon examination and previous history were primary drivers of handling approaches. Though almost all participants initially used minimal or standing restraint, signs of fear or aggression prompted them to pause and make alternate handling decisions. In such situations, many opted to (1) apply a muzzle (fabric or basket), towel or blanket wrap, (2) administer sedation, (3) ask the owner to reschedule the examination with pre-visit medications, and/or (4) have technicians or other support staff assist with restraining dogs in the examination room or in another area of the clinic away from the owner (e.g., treatment room). Participants expressed varied perspectives about the use of certain restraint techniques; for example, some felt chemical restraint was necessary for preventing safety risks, while others felt it could impair diagnostic accuracy. Participants also described strategies for adapting handling in response to dog behavior, particularly fear and aggression, with decisions shaped by contextual factors, such as safety concerns and divergent views on owner presence during examinations. Further, participants did not describe the use of full-body restraint, despite evidence from a North American cross-sectional survey indicating that this approach is commonly used by veterinary staff when handling fearful or aggressive dogs during routine care (19). Although it is possible that participants use higher levels of restraint in practice but did not discuss them during interviews, this absence may also reflect deliberate avoidance of an aversive handling technique. In contrast, participants also did not describe the use of certain behavior modification techniques, including counterconditioning or desensitization, despite these being recommended to reduce dog stress and improve welfare during veterinary care (6, 12). Participants mentioned the provision of treats, particularly in the context of creating distractions and/or positive associations; however, not in a manner that reflects the practice of counterconditioning, where the examination is broken down into small approximations, and pairing each step with a reward (6, 12). While it is possible that these strategies are used in practice but were not explicitly discussed, this may instead suggest limitations in performing this in practice (e.g., time constraints), or a lack of awareness and education among practitioners about behavior modification techniques, highlighting an important area for future research.

Safety was widely discussed as a top priority, with several participating veterinarians expressing concerns about bite risk to owners and associated clinic liabilities. To reduce this risk, several preferred to take the dog to the treatment area to be handled by technicians as they felt that the owner’s presence provoked ‘protective tendencies.’ Several explained that some dogs ‘do better’ when only veterinary staff are involved as they felt that owners can complicate handling (e.g., made attempts to handle their dog), whereas others preferred owner presence as they felt this improved dog comfort and cooperation (e.g., providing treats, petting, verbal reassurance, assisting with restraint). Though no research has evaluated how owner presence influences safety during clinical examinations, there are mixed findings on the impact of owner presence, where it has been reported to improve behavior (20, 42, 43), or increase dog stress (44, 45). Despite this, owners are encouraged to remain present during physical examinations, when possible, to reduce dog stress (6, 12).

Additional strategies reported to maintain safety for staff, patients, and pet owners involve using muzzles, towels or blanket wraps, sedation, or rescheduling the examination with pre-visit medications. Many participants also emphasized the importance of preventive strategies to reduce dog fear, such as encouraging owners to engage in cooperative care training and scheduling ‘happy visits’ (bringing their dog to the clinic just for treats). However, client compliance with this was highly variable. Further, several participants cited costs and additional time associated with certain stress-reducing practices, such as rescheduling appointments with pre-visit medications or using injectable sedation, as deterrents for some owners. Future research should explore barriers to client compliance with veterinarian recommendations and use of certain stress-reducing practices (e.g., pre-visit medications), as this may help overcome this common challenge and improve appointments.

Clinic factors, such as operational policies, staffing, management, shared values (e.g., attitudes towards stress-reducing practices), and efficiency pressures, were also commonly reported as either conducive or prohibitive to applying stress-reducing practices. This parallels findings from previous survey studies which have identified high caseloads, time pressure, staffing constraints, clinic structure, costs, and workplace culture as pervasive barriers to stress-reducing practices (17, 18, 46). In the present study, time pressure (e.g., due to high caseloads and limited appointment lengths) resulted in some participants not applying certain stress-reducing practices. For example, during high caseloads, some participants described not pausing or stopping an examination if a dog showed signs of fear. A similar pattern was reported by Nakonechny et al. (19), who found that staff who felt constrained by appointment time were more likely to use full-body restraint on dogs, suggesting that time pressure can shift handling toward techniques that prioritize efficiency over patient comfort. Limited research is available on the efficiency of appointments where stress-reducing practices are used. However, one experimental study found that stress-reducing interventions increased appointment times by 2.5 min for dog examination times across four visits (13, 14). Though this reflects increased appointment length, the additional time may be negligible in practice settings, especially given the benefits it can provide for dog welfare, client satisfaction, and safety. For example, a survey study reported reduced patient-induced injury rates (e.g., bites, scratches) among companion animal clinics where all staff had a stress-reducing certification (16). In addition to enhanced safety, several participants expressed that clients often appreciated extra time spent making their dog comfortable and communicating with them during the appointment.

The training, previous experience, and perceptions of the veterinary team shaped handling choices, with limited “buy-in” and insufficient training frequently described as barriers to stress-reducing practices. Many participants reported minimal exposure in veterinary school (including internships and rotations) to fearful or aggressive dogs and little instruction on appropriate handling techniques or tools and stress-reducing practices, often encountering mentors who modeled ‘old-school’ approaches, emphasizing examination or procedure completion over the animal’s experience. These accounts align with variability in curricula and gaps in behavior education: only 73% of US veterinary schools reported a formal animal behavior course (47), and fewer than half of American veterinarians perceived their animal behavior training as adequate, with 39% reporting only a few hours of lectures and 18% had none (48). Cultural and structural impediments persisted, such as colleagues perceiving stress-reducing methods to be time-consuming or costly, having limited managerial support, and participants relying on *ad hoc* learning via colleagues and continuing education. This suggests the need for future research aimed at developing and evaluating evidence-based behavior change interventions and curriculum enhancements to improve stress-reducing training in veterinary education, coupled with culture change and managerial support among clinics for consistent implementation.

Almost all participants described experiencing burnout at some point during their career, particularly during their first years of clinical practice or when working at high-volume or emergency practices, and long hours. Several explained that they felt experiencing burnout or secondary traumatic stress did not affect their interactions with animal patients, citing effective coping strategies, such as compartmentalization, as being effective in managing their demeanor between appointments. Conversely, several felt that experiencing burnout and/or secondary traumatic stress negatively impacted their interactions with clients or patients. Specifically, they felt they were less patient (e.g., rushing through appointments), and willing to take time to communicate with clients. Further, some said that they did not perform certain stress-reducing practices (e.g., provision of treats, pausing or stopping the examination if a dog shows fear) when experiencing burnout or high levels of stress. This was similarly reported in a Canadian qualitative study by Campbell et al. (25), where some veterinarians described decreased quality of communication and patience with clients, and doing a “quick, short examination” rather than fully assessing patients, when experiencing high levels of stress or poor mental health. These findings align with the ‘One Welfare’ framework, which recognizes the interconnectedness between animal welfare, human well-being, and the physical and social environment (49). In this context, reported declines in stress-reducing handling practices, more rushed and less thorough examinations, and less communication during periods of burnout suggest that practitioner well-being may influence the quality of animal–human–client interactions.

### Limitations

This study provides novel insights into veterinarians’ perspectives on handling decisions and stress-reducing practices during routine dog examinations; however, several limitations should be acknowledged. Though the findings of this study may not be generalized to the broader veterinarian population, the purpose of these interviews was to provide in-depth insights about the handling approaches that veterinarians use and what factors shape their decisions. The sample comprised of 17 veterinarians practicing in Canada and the United States, recruited primarily through social media and professional networks. While this approach facilitated access to a diverse range of participants across regions and practice types, it may also have led to self-selection bias, as individuals with a particular interest in stress-reducing practices may have been more likely to participate. Further, the sample was disproportionately comprised of veterinarians who identified as female, which may limit transferability of the findings. Although most veterinarians practicing in Canada and the United States identify as female (50, 51), the present study may underrepresent handling approaches more commonly used by male veterinarians.

Although inductive QCA allowed for an in-depth exploration of an under-researched topic, the analytic process is inherently interpretive. While steps, such as peer debriefing and maintaining an audit trail, were taken to enhance credibility and dependability, the findings reflect the perspectives of participants as well as the interpretive lens of the investigators (52). Future studies could strengthen confirmability by incorporating additional strategies such as multiple coders, participant validation (member checking), or triangulation with observational data (52). During transcription, each transcript was assessed for accuracy. Where needed, grammatical corrections (e.g., adding periods or commas) were made, and inaccurate words were adjusted. Although these corrections have potential to alter the meaning of some sentences (32) the investigator attempted to minimize this risk by simultaneously listening to the audio recordings while reviewing the transcripts. The interviews relied on self-report, which may be influenced by recall or social desirability biases (53). Participants may have underreported restrictive handling practices or overemphasized the use of stress-reducing strategies. Observational and longitudinal research examining actual handling practices in clinical settings would provide valuable complementary data to assess alignment between reported and enacted behaviors.

While this study focused on routine physical examinations, many veterinarians highlighted situational factors, such as the urgency of care, to influence their handling. Future research should investigate contextual influences more systematically, particularly in emergency practices or other environments (e.g., high volume spay/neuter clinics, shelter medicine) where stress-reducing practices are perceived to be more challenging to implement. Similarly, further studies could examine how organizational culture and workplace resources shape the feasibility of stress-reducing practices, and affect compassion fatigue and compassion satisfaction, especially among those who value stress-reducing practices. By addressing these gaps, future work can contribute to practical strategies that support dog welfare and the well-being of veterinarians.

## Conclusion

These findings show how veterinarians navigate handling decisions during dog physical examinations, which are contextual and influenced by patients, clients, and clinical environments. While participants valued and commonly used stress-reducing practices, many described practical constraints that influenced when and how these practices were applied, such as time pressure, staffing, space limitations, and lack of widespread clinic adoption. Taken together, the findings highlight that effective implementation of stress-reducing handling requires not only individual education and training, but multi-level strategies. Beyond individual training, clinic policies, appointment structures, staffing, leadership priorities, and owner expectations all influence how dogs are handled. Involving stakeholders such as veterinarians, technicians, clinic managers, professional bodies, and dog owners in co-developing realistic protocols may support more sustainable change. The findings of this study also point to a bidirectional relationship between dog handling and clinician well-being: using stress-reducing approaches may promote better clinical care and compassion satisfaction, whereas compassion fatigue can diminish patience and consistency in handling. Future research should build on these insights by using observational and mixed-methods designs to compare reported versus enacted handling approaches, evaluate the efficiency and safety outcomes of stress-reducing protocols, and assess how these strategies function across diverse practice environments (e.g., high-volume, emergency, or shelter settings).

## Data Availability

The raw data supporting the conclusions of this article will be made available by the authors, without undue reservation.
