# Supplementary material for: In their own words: qualitative interviews with veterinarians on handling decisions during dog examinations
Source: Front Vet Sci. 2026 Mar 20;13:1761014. doi: 10.3389/fvets.2026.1761014 (PMC13046485; doi:10.3389/fvets.2026.1761014)
Supplement: Supplementary file 1 [file Table_1.docx]

Supplementary Material

**Interview Guide:**

**Q1:** From the questionnaire that you completed, I saw that you are a [insert practice type] practitioner and you indicated that you work in a [clinic/mobile clinic]; so just to confirm, you would typically perform appointments in an [exam room/in someone’s home]. Can you walk me through your typical routine exam appointment with a dog patient, from start to finish?

Probes:

1. What do you do when you first enter the exam room? For example, do you approach the dog right away or at what point do you begin interacting with them?
2. How do you like to set up your exam room or area?
3. Where do you typically perform an exam and why? (e.g., exam table, on the ground).
4. Does anything in particular affect where you choose to examine the dog? For example, the size of the dog or how the dog is behaving.
5. Do you do any animal handling, or do you have veterinary technicians or assistants who perform most of the handling?

**Q2:** Are there any particular factors about a dog that affect how you interact with them during an appointment?

Probes:

1. For instance, some factors could be the dog’s size, behavior, or breed.

**Q3:** In a recent survey study that our research team completed, we found that an owner being present during an exam influenced how veterinarians handle dogs. Would you say this is also true for you, and why or why not?

Probes:

1. Do you prefer that an owner is present or absent during an exam? Why or why not?
2. How does the owner’s presence, if at all, change how you conduct the exam?
3. How do you feel when you need to restrain a dog with the owner present?
4. How do you think pet owners feel when you restrain their dog during an exam?

**Q4:** Thinking back to your time in vet school, what can you recall about your training in handling fearful or aggressive dogs? Examples of fear would include things such as a dog with a lowered posture or tucked tail, shaking, or lip licking. Examples of aggression in dogs could include growling, baring teeth or attempting to bite.

Probes:

1. What handling techniques were you taught to use in vet school when a dog is showing fear or aggression, or has a known history of being fearful or aggressive?
2. Were the dog handling techniques that you learned in your coursework different from what you used in your clinical rotations? If so, how?

**Q5:** In your current practice, can you tell me about what you typically do when a dog is showing fear or aggression during an appointment?

Probes:

1. For example, what actions, handling techniques, or types of equipment might you use in these situations?
2. What do you find most challenging when handling a fearful or aggressive dog?
3. Are there any specific reasons why you choose to use these types of handling techniques?

**Q6:** How do you feel emotionally when a dog is showing fear or aggression during an appointment?

**Q7:** Do you have any memorable appointments or experiences with a dog that has changed how you currently approach or handle dogs?

Probes:

- Have you had any negative experiences with a dog patient, such as a bite injury, that has influenced how you handle dogs currently?
- Have you had any positive experiences with a dog patient that has influenced how you handle dogs currently?

**Q8:** What are your thoughts on the low-stress handling philosophy of dogs during veterinary care?

And just so we are on the same page, when I say low-stress handling, I’m referring to handling techniques that involve assessing the dog’s behavior and applying the least amount of restraint to conduct an exam.

Probes:

**If they need further clarification on low-stress handling techniques:**

1. Fear Free Pets is an example of an organization that promotes the use of low-stress handling. These techniques aim to reduce fear, stress, and anxiety in dogs, and could include things such as providing positive rewards or distractions for dogs during an exam, using minimal restraint, and releasing dogs from a restraint hold if they start to escape from the restraint.

**If they use low-stress handling techniques**:

1. What benefits have you noticed from using low-stress handling techniques?
2. Are there any challenges you face when using low-stress handling techniques?

**If they do not use low-stress handling techniques:**

1. Are there any specific reasons why you are not inclined to use low-stress handling techniques in practice?

For the next few questions, I’d like to discuss a concept called professional quality-of-life (ProQOL). ProQOL refers to the positive and negative aspects of working as a professional caregiver, which includes veterinary work. There’s a positive aspect—referred to as compassion satisfaction, and two negative aspects, which are burnout and secondary traumatic stress.

I’ll just drop an infographic in the chat, so you have a visual of this concept as we go through the next few questions [drop infographic in chat].

Compassion satisfaction refers to feelings of pleasure and fulfillment that you get being able to do your work well as a veterinarian. Burnout refers to physical, emotional, and mental exhaustion, and it may include feelings of hopelessness and a sense of being overwhelmed.

And lastly, secondary traumatic stress refers to emotional distress from exposure to trauma experienced by others. So, for example, in your work, you may experience secondary traumatic stress from things such as seeing your dog patients in significant pain, or clients being emotionally distressed about their pet.

**Q9:** What, if any, experiences have you had with burnout during your veterinary career?

- **If yes:**
  - Do you think experiencing burnout has had any impact on how you interact with your patients?
- **If no:**
  - What factors or strategies do you think have helped you avoid burnout?
  - If you did experience burnout, how do you think this might affect how you interact with your patients, if at all?

**Q10:** Have you ever felt highly distressed or traumatized after working with an animal, or from witnessing a distraught pet owner?

- **If yes:**
  - Has this experience affected how you interact with your patients? If so, how so?
- **If no:**
  - If you did experience secondary traumatic stress, how do you think this might affect how you interact with your patients, if at all?

**Q11:** Do you feel that you experience compassion satisfaction (or pleasure and fulfillment) from your work as a veterinarian?

- **If yes:**
  - What experiences or moments in your work bring you compassion satisfaction?
  - How do you think compassion satisfaction affects how you interact with your patients?
- **If no:**
  - If you did experience compassion satisfaction, how do you think this would affect how you interact with your patients, if it all?
