# Supplementary material for: In their own words: qualitative interviews with veterinarians on handling decisions during dog examinations
Source: Front Vet Sci. 2026 Mar 20;13:1761014. doi: 10.3389/fvets.2026.1761014 (PMC13046485; doi:10.3389/fvets.2026.1761014)
Supplement: Supplementary file 2 [file Table_2.docx]

**Supplementary File 2. Summary of the categories, sub-categories, and codes generated from the content analysis of interviews with veterinarians (n=17).**

| Categories and sub-categories | Codes |  |
| --- | --- | --- |
| Category 1: Approaches to dog handling | |  |
| 1. Stress-reducing practices | a. Environmental modifications  b. Adapting handling strategies  c. Positive experiences |  |
| 1. Restraint techniques and tools | a. Restraint techniques  b. Restraint tools |  |
| 1. Examination conduct and procedures | a. Examination location  b. Procedures and documentation  c. Personnel involved with handling |  |
| Category 2: Factors affecting dog handling | |  |
| 1. Dog patients | a. Dog behavior  b. Dog pain and health  c. Dog characteristics |  |
| 1. Dog owners | a. Owner-veterinarian interactions  b. Owner presence  c. Owner expectations and preferences |  |
| 1. Workplace | a. Decision-making autonomy  b. Time and efficiency pressures  c. Clinic resources and constraints  d. Team training and alignment |  |
| 1. Formative experiences and training | a. Veterinary school  b. Work experiences  c. Personal experiences  d. COVID-19 pandemic |  |
| Category 3: Veterinarian professional well-being | |  |
| 1. Compassion fatigue | a. Sources of compassion fatigue  b. Impacts of compassion fatigue  c. Coping with compassion fatigue |  |
| 1. Compassion satisfaction | a. Sources of compassion satisfaction  b. Impacts of compassion satisfaction |  |
| Category 4: Perceptions of stress-reducing practices for patients | | |
| 1. Perceived benefits | | a. One Welfare  b. Ease of dog handling and safety  c. Increased productivity |
| 1. Perceived challenges | | a. Lack of support and resources  b. Decreased efficiency |
